# Lipoprotein(a) in Coronary Artery Disease and Aortic Stenosis: Pathophysiology, Clinical Impact, Interventional Implications and Emerging Targeted Therapies

**DOI:** 10.3390/jcm15155897

**Published:** 2026-07-28

**Authors:** Francesco Maria Animati, Simone Proietti, Francesco Auletta, Rocco Antonio Montone, Luigi Cappannoli, Francesco Fracassi, Achille Gaspardone, Francesco Burzotta

**Affiliations:** 1 Dipartimento di Scienze Cardiovascolari e Pneumologiche, Università Cattolica del Sacro Cuore, 00168 Rome, Italy; 2 Department of Cardiovascular Medicine—CUORE, Fondazione Policlinico Universitario A. Gemelli IRCCS, 00168 Rome, Italy; 3 Division of Cardiology, Sant’Eugenio Hospital, 00144 Rome, Italy

**Keywords:** lipoprotein(a), percutaneous coronary intervention, residual risk, transcatheter aortic valve implantation, coronary inflammation

## Abstract

Lipoprotein(a) (Lp(a)) is increasingly recognized as a genetically determined and clinically relevant contributor to residual cardiovascular risk. Through pro-atherogenic, pro-inflammatory, pro-thrombotic, and pro-calcific mechanisms, Lp(a) appears to play a significant role in both coronary artery disease and aortic valve disease. In patients undergoing percutaneous coronary intervention, elevated Lp(a) has been associated with worse long-term outcomes, including recurrent ischemic events, repeat revascularization, and in-stent restenosis, even in the setting of controlled low-density lipoprotein cholesterol. In parallel, experimental, genetic, and clinical data support a role for Lp(a) in the initiation and progression of calcific aortic stenosis, while its prognostic significance after transcatheter aortic valve interventions remains less clearly defined. Current guidelines now recognize Lp(a) as a relevant risk-enhancing factor, and emerging targeted therapies are achieving substantial reductions in circulating levels. Overall, Lp(a) should be regarded as both a meaningful biomarker and a promising therapeutic target, although ongoing outcome trials are needed to determine whether selective Lp(a) lowering translates into clinical benefit across interventional cardiovascular settings. The aim of this narrative review is to provide a single, comprehensive account of lipoprotein(a) [Lp(a)] in interventional cardiology, following this lipoprotein from its biology and pathophysiology through to its clinical impact and to the therapies that are now reaching the clinic, with a specific focus on the two most frequent catheter-based procedures in which it may carry prognostic weight: percutaneous coronary intervention (PCI) and transcatheter aortic valve implantation (TAVI). PCI and TAVI are deliberately addressed within the same review since they share a common upstream biology: Lp(a) contributes both to the atherosclerotic process that underlies coronary disease and to the calcific process that underlies aortic valve disease, and the corresponding patient populations overlap considerably in everyday interventional practice. Covering them together offers the interventional cardiologist a single, practical reference on how a patient with elevated Lp(a) may be approached in both scenarios.

## 1. Introduction

Since its initial description in 1963 by Berg and colleagues [1] as an antigenic determinant associated with low-density lipoprotein, lipoprotein(a), (Lp(a)) has been recognized as a distinct lipoprotein entity composed of an LDL-like particle and apolipoprotein(a), a highly glycosylated protein of variable molecular mass covalently attached to apolipoprotein B-100 [2]. Only nine years later, the same research group linked Lp(a) for the first time with a higher risk for the development of coronary artery disease (CAD) [3]. A major turning point in Lp(a) research came in 1987, when apolipoprotein(a) was found to share a marked structural similarity with plasminogen, the central zymogen of the fibrinolytic system [4]. In the contemporary era, a growing body of evidence consistently links elevated Lp(a) levels with a higher prevalence of atherosclerotic cardiovascular disease (ASCVD), coronary artery disease (CAD), calcific aortic valve stenosis, and cerebrovascular events, with this association persisting even after adjustment for traditional risk factors [5]. Notably, high Lp(a) concentrations have also been associated with refractory angina, further supporting its potential role not only in atherothrombotic risk but also in clinically relevant symptom burden among patients with coronary artery disease [6,7].

The incidence of major adverse cardiovascular and cerebrovascular events (MACCE) demonstrates a positive correlation with Lp(a) concentrations in both primary and secondary prevention cohorts [8]. However, the precise morphology of this dose–response relationship appears to be modulated by the patient’s underlying risk profile. Specifically, while the risk trajectory follows a largely linear pattern in primary prevention, it exhibits greater complexity in secondary prevention settings, where even relatively modest elevations in Lp(a) can exert a profound clinical impact [9]. Rather than acting as a passive diagnostic indicator, Lp(a) functions as a potent “risk intensifier” that scales the absolute probability of cardiovascular complications [10]. This paradigm is especially critical for individuals with documented CAD; in these patients, an already high intrinsic vulnerability to recurrent ischemic episodes is further exacerbated by the presence of elevated Lp(a) [10]. In summary, elevated Lp(a) represents a critical component of residual cardiovascular risk, linked to extensive baseline CAD, severe aortic stenosis, and recurrent in-stent restenosis. Since standard lipid-lowering therapies fail to address this genetically determined particle, understanding its prognostic impact is essential for the next generation of targeted cardiovascular interventions [11].

### 1.1. Lp(a) Pathophysiology

**Figure 1:**
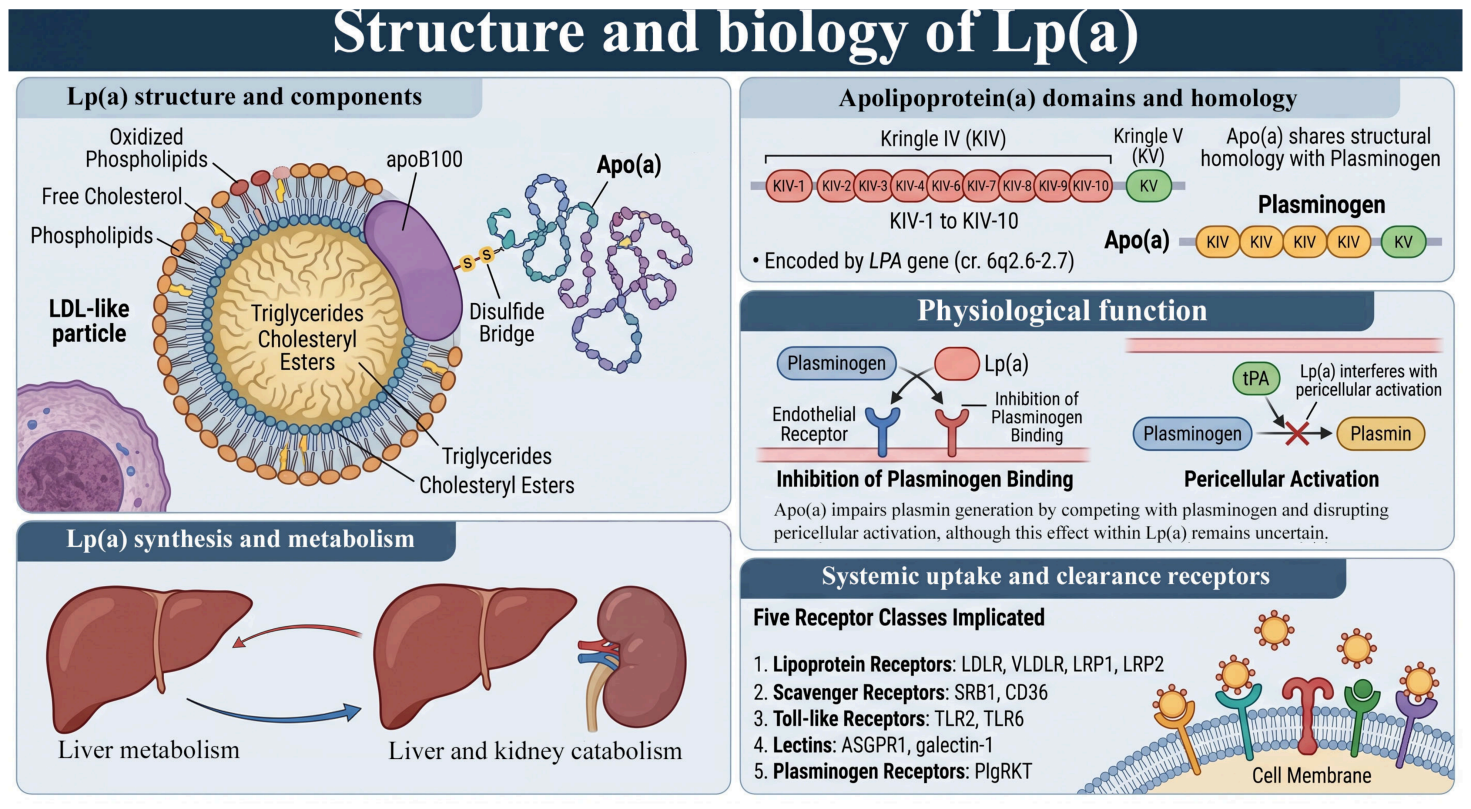
Structural composition, metabolism, and biological function of Lipoprotein(a). The Lp(a) particle consists of an LDL-like core covalently bound to apolipoprotein(a) via a single disulfide bridge. Due to the high structural homology between the Kringle domains of Apo(a) and plasminogen, Lp(a) competitively inhibits plasminogen binding and pericellular activation, thereby impairing fibrinolysis. The figure further illustrates the hepato-renal pathways of Lp(a) synthesis and catabolism, alongside the diverse, multi-class membrane receptors mediating its systemic cellular uptake. Abbreviations: Apo(a): Apolipoprotein(a), ApoB–100: Apolipoprotein B–100, ASGPR1: Asialoglycoprotein Receptor 1, KIV: Kringle IV, KV: Kringle V, LDL: Low-Density Lipoprotein, LDLR: Low–Density Lipoprotein Receptor, Lp(a): Lipoprotein(a), LRP1: LDL Receptor-Related Protein 1, LRP2: LDL Receptor-Related Protein 2, PlgRKT: Plasminogen Receptor (KT), SRB1: Scavenger Receptor Class B Type 1, TLR2/6: Toll-Like Receptor 2/6, tPA: Tissue Plasminogen Activator, VLDLR: Very Low-Density Lipoprotein Receptor.

- Structure and biology of Lp(a) (Figure 1)

Lp(a) is a macromolecular complex consisting of a low-density lipoprotein cholesterol (LDL)-like particle containing apoB100 covalently linked via a disulfide bridge to apolipoprotein(a), encompassing a core of triglycerides, cholesteryl esters, and oxidized phospholipids [12]. Apo(a), a glycoprotein encoded by the LPA gene (cr. 6q2.6–2.7), shares structural homology with plasminogen due to the presence of specific protein domains termed Kringle IV (KIV) (classified into subtypes 1–10) and Kringle V (KV) [13].

The physiological function of Lp(a) remains elusive. Mounting evidence suggests that Apo(a) may inhibit the conversion of plasminogen to plasmin, both by competing with plasminogen for endothelial receptors [14] and by interfering with pericellular activation mechanisms [15]; however, it remains unclear whether this role is preserved when Apo(a) is integrated into the Lp(a) structure [15].

Lp(a) is synthesized in the liver [16], and its metabolism occurs independently of other lipoproteins [17]. The hepatic synthesis of lipoprotein(a) is a highly complex, five-stage process [18]. The LPA gene is transcribed into a stable mRNA transcript within the hepatocyte nucleus, which is subsequently translated into apolipoprotein(a) at the ribosomes [18,19,20]. Nascent apo(a) undergoes initial post-translational modifications within the endoplasmic reticulum (ER)—including the formation of three disulfide bonds and the addition of N-linked glycans to the apo(a) Kringle domains—both of which are critical for proper protein folding [18,21,22]. Apo(a) is then further processed through the Golgi apparatus [23] before being trafficked to the hepatocyte plasma membrane [24]. The assembly mechanism of Lp(a) proceeds via a distinct two-step sequence [18]. Initially, a non-covalent interaction is established between specific KIV domains (mostly, KIV3–6) and the lysine groups of apolipoprotein B100 [25]. This is followed by the formation of a definitive, stable disulfide bond between the KIV9 domain of apo(a) and apoB100 of the LDL component [18,26,27]. To date, a clear consensus regarding the exact site of Lp(a) assembly remains elusive in the literature. While some authors suggest that assembly occurs at the hepatocyte plasma membrane or within the space of Disse [28], others contend that it takes place directly in the plasma or the interstitial space [29]. Catabolism occurs primarily in the liver and, to a lesser extent, in the kidneys [30]. Conversely, the molecular mechanisms underlying its systemic uptake and clearance remain poorly defined. Five receptor classes are implicated in the plasma uptake of Lp(a): lipoprotein receptors (LDLR, VLDLR, LRP1, LRP2), scavenger receptors (SRB1, CD36), Toll-like receptors (TLR2, TLR6), lectins (ASGPR1, galectin-1), and plasminogen receptors (PlgRKT) [31]. While low-density lipoprotein cholesterol receptors (LDLRs) are nominally involved in Lp(a) catabolism, it must be noted that conflicting evidence remains concerning their role in this process [32,33,34,35,36,37,38]; furthermore, statins—which act by upregulating LDLRs expression—do not reduce serum Lp(a) concentrations [39].

- Differences across populations

Lp(a) levels are 70–90% genetically determined and depend on the codominant expression of the two LPA alleles [40]. Specifically, the majority of this genetic variability is attributed to a copy number variation (CNV) polymorphism in KIV-2 [41]. Since smaller apo(a) isoforms are associated with a more rapid exit of the apo(a) precursor from the endoplasmic reticulum (ER), an inverse correlation exists between circulating Lp(a) levels and the number of KIV-2 repeats [42,43,44]. Although the association between apo(a) isoform size and serum Lp(a) concentrations is well established, this relationship is non-linear, and substantial variability in serum Lp(a) values remains even among individuals carrying the same isoform [45,46]. To a lesser extent, single-nucleotide polymorphisms (SNPs) at other LPA polymorphic loci, as well as polymorphisms within the APOEε2 and APOH genes, also modulate serum Lp(a) levels [47,48,49]. Evidence regarding the impact of epigenetics on LPA gene expression remains limited. In an epigenome-wide analysis, Coassin et al. identified a CpG site within the LPA promoter; however, statistical mediation analyses demonstrated that the effect on apo(a) synthesis stems from an underlying SNP (rs76735376) and the subsequent nucleotide substitution, rather than direct methylation of the locus [50]. Similarly, while the role of certain cytokines, such as IL-6, in regulating serum Lp(a) levels is well recognized [51], evidence supporting the presence of potential epigenetic regulatory mechanisms is still lacking.

Although playing a secondary role, non-genetic factors also contribute to determining serum Lp(a) levels. Low-carbohydrate and high-saturated-fat dietary patterns, and diets rich in walnuts or pecans, have been shown to reduce serum Lp(a) levels by approximately 15% and 6–15%, respectively [52]. Similarly, postmenopausal hormone replacement therapy (HRT) has been demonstrated to produce statistically significant reductions in serum Lp(a) levels [53,54]. Finally, certain pathological conditions, such as hypothyroidism or chronic kidney disease (including hemodialysis, peritoneal dialysis, and nephrotic syndrome), have been associated with increased serum Lp(a) concentrations [55,56,57,58], whereas hyperthyroidism and hepatocellular damage have been linked to reduced serum Lp(a) levels [56,59,60,61]. Of note, environmental factors, particularly air pollution and elevated PM2.5 levels, have also been associated with increased lipid levels and higher Lp(a) concentrations in exposed patients [62,63].

**Figure 2:**
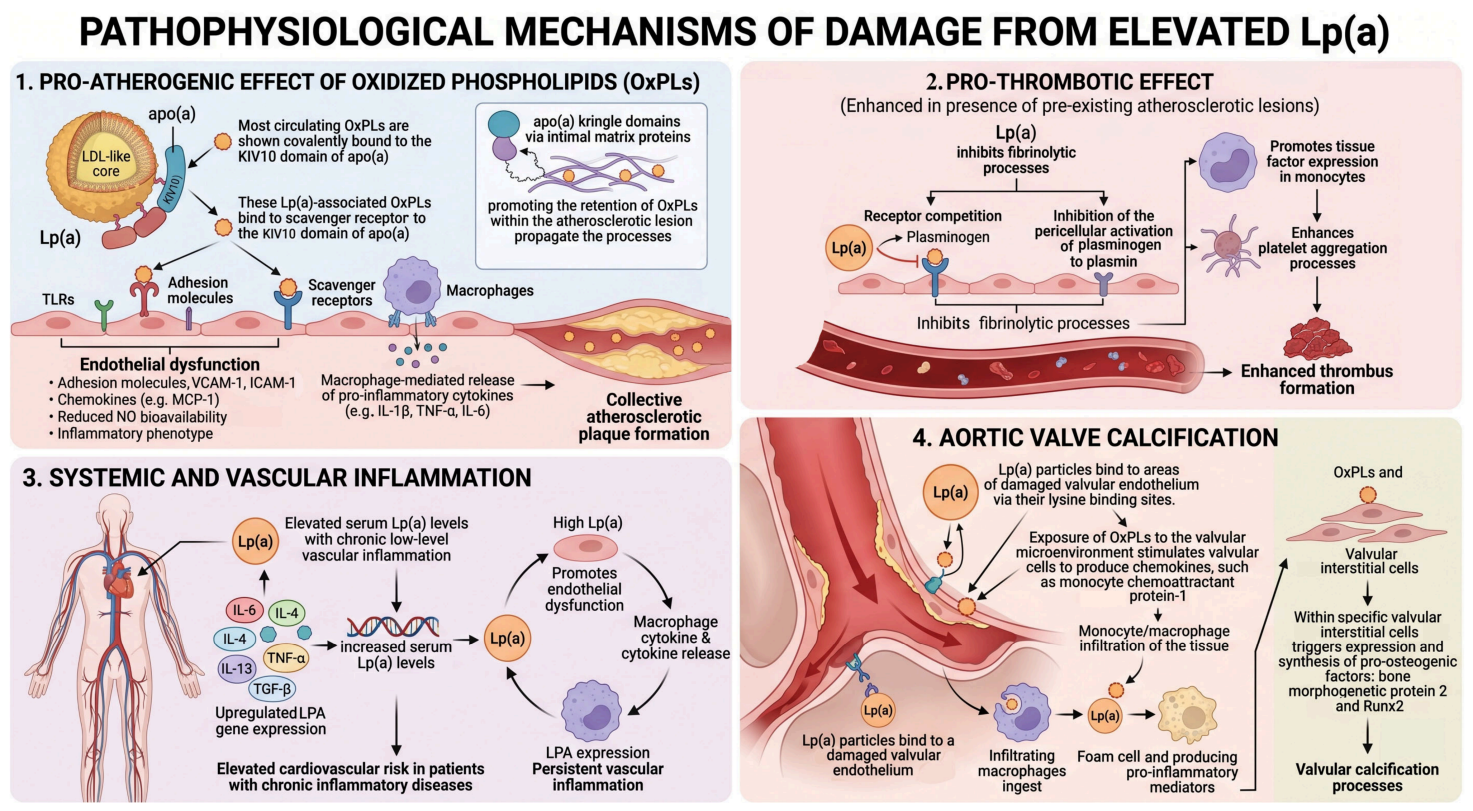
Primary pathophysiological mechanisms mediating cardiovascular damage from elevated Lipoprotein(a). These pleiotropic pathways include: (1) pro-atherogenic effects driven by Lp(a)-associated oxidized phospholipids, promoting endothelial dysfunction and macrophage activation; (2) pro-thrombotic activity resulting from competitive inhibition of fibrinolysis and enhanced platelet aggregation; (3) systemic and vascular inflammation perpetuated by chronic cytokine release; and (4) promotion of aortic valve calcification through macrophage infiltration and osteogenic differentiation of valvular interstitial cells. Abbreviations: Apo(a): Apolipoprotein(a), ICAM-1: Intercellular Adhesion Molecule 1, IL: Interleukin (IL-1β, IL-4, IL-6, IL-13), KIV: Kringle IV, LPA: Lipoprotein(a) gene, Lp(a): Lipoprotein(a), MCP-1: Monocyte Chemoattractant Protein-1, NO: Nitric Oxide, OxPLs: Oxidized Phospholipids, Runx2: Runt-related transcription factor 2, TGF-β: Transforming Growth Factor Beta, TLRs: Toll-Like Receptors, TNF-α: Tumor Necrosis Factor Alpha, VCAM-1: Vascular Cell Adhesion Molecule 1, VICs: Valvular Interstitial Cells.

- Pathophysiological mechanisms of damage (Figure 2)

Elevated Lp(a) levels contribute to cardiovascular disease through four distinct mechanisms: the pro-atherogenic effect of oxidized phospholipids, pro-thrombotic activity, systemic and vascular inflammation, and aortic valve calcification [64].

(A) Pro-atherogenic effect of oxidized phospholipids

Most circulating oxidized phospholipids (OxPLs) within Lp(a) are covalently bound to the KIV10 domain of Apo(a) [65]. Lp(a)-associated OxPLs bind to TLRs and scavenger receptors on endothelial cells and macrophages [66], contributing to endothelial dysfunction—characterized by adhesion molecule and chemokine expression, reduced nitric oxide bioavailability, and an inflammatory phenotype [67,68]—and the macrophage-mediated release of pro-inflammatory cytokines [69,70,71,72]. These events collectively drive atherosclerotic plaque formation [73]. Furthermore, Apo(a) Kringle domains can bind to intimal matrix proteins via their lysine binding sites [64,74,75], promoting the retention of OxPLs within the atherosclerotic lesion and propagating the aforementioned processes [18].

(B) Pro-thrombotic effect

Lp(a) exerts pro-thrombotic effects primarily in the presence of pre-existing atherosclerotic lesions [64]; indeed, elevated Lp(a) levels are not associated with an increased risk of deep vein thrombosis [76]. Lp(a) can inhibit fibrinolytic processes both through receptor competition with plasminogen [14] and by inhibiting the pericellular activation of plasminogen [15]. Similarly, evidence suggests that Lp(a) may promote tissue factor expression in monocytes and enhance platelet aggregation processes [64,77].

(C) Systemic and vascular inflammation

Elevated serum Lp(a) levels are associated with chronic low-level vascular inflammation [64,72,78]. Various cytokines, including IL-6, IL-4, IL-13, TNF-α, and TGF-β, upregulate LPA gene expression [79,80,81,82], leading to increased serum Lp(a) levels. This effect likely contributes to the elevated cardiovascular risk observed in patients with chronic inflammatory diseases [83,84]. Additionally, high Lp(a) levels promote endothelial dysfunction [67,68] and macrophage cytokine release [69,70,71,72], triggering a vicious cycle that leads to further LPA expression and persistent vascular inflammation [85].

(D) Aortic valve calcification

Lp(a) binds to areas of damaged valvular endothelium via its lysine binding sites [86]. Exposure of OxPLs to the valvular microenvironment stimulates valvular cells to produce chemokines—such as monocyte chemoattractant protein-1 (MCP-1)—promoting monocyte/macrophage infiltration of the tissue [64,87]. Infiltrating macrophages subsequently ingest Lp(a) particles, transforming into foam cells and producing pro-inflammatory mediators [88]. Within this specific cytokine milieu, the interaction between OxPLs and valvular interstitial cells (VICs) triggers the expression and synthesis of pro-osteogenic factors, such as bone morphogenetic protein 2 (BMP2) and Runx2 [89], which underpin valvular calcification processes [90].

### 1.2. The Role of Lp(a) in Coronary Artery and Cerebrovascular Disease

The pathophysiological role of Lp(a) in coronary artery disease is not fully understood yet. The mechanisms that have been proposed include a pro-atherogenic, pro-thrombotic, and pro-inflammatory pathways [91]. Despite the inconclusive nature of the existing evidence, it has already been demonstrated that increased Lp(a) concentrations are independently linked to a higher risk of developing coronary artery disease [92]. In a 1998 meta-analysis, Craig et al. reported that Lp(a) concentrations were elevated in subjects who subsequently developed CAD compared with those who did not [93]. By extending the meta-analysis with a further 10 years of follow-up, Danesh et al. confirmed a clear association between Lp(a) and CAD, including 5436 additional deaths from CAD [94]. More recently, the INTERHEART study investigated the contribution of Lp(a) concentration to myocardial infarction risk in the general population, showing that Lp(a) levels > 50 mg/dL were linked to an increased risk of myocardial infarction, with higher burden in South Asians and Latin Americans [95]. Moreover, it has been demonstrated that Lp(a) levels are associated with higher CAD risk independently of LDL-C levels. Furthermore, in a recent, large participant-level meta-analysis of placebo-controlled statin trials, Lp(a) and low-density lipoprotein cholesterol emerged as independent and additive determinants of atherosclerotic cardiovascular risk [96]. Elevated Lp(a) was associated with a higher incidence of cardiovascular events irrespective of achieved low-density lipoprotein cholesterol levels, indicating that low-density lipoprotein cholesterol reduction alone does not fully abolish Lp(a)-related residual risk. Overall, these findings reinforce the concept that Lp(a) confers incremental risk beyond conventional lipid parameters [96].

Beyond its established pro-atherogenic and pro-inflammatory properties, Lp(a) has also been implicated in the development and progression of coronary artery calcification (CAC) [97,98]. In a primary prevention cohort study by Burzynska et al. conducted in patients undergoing coronary computed tomography angiography, higher Lp(a) concentrations were associated with a greater coronary calcific burden as measured by CAC score. Moreover, patients with higher Lp(a) tended to show progressively higher CAC values, with CAC increasing in parallel with incremental rises in Lp(a). Importantly, lower Lp(a) levels were associated with a higher probability of having CAC = 0, supporting a potential “power of zero” concept in patients without significant Lp(a)-related risk amplification [99,100]. In line with this, a recent meta-analysis by Martignoni et al. has shown that in asymptomatic patients elevated Lp(a) was linked to a 31% increase in the likelihood of detectable CAC, a 29% increase in the likelihood of more advanced CAC, and a 43% increase in the likelihood of CAC progression during follow-up. In addition, the probability of showing coronary calcification rose progressively with increasing Lp(a), with an estimated 1% increase for each 1 mg/dL increment [101].

As for ischemic stroke risk, in a large contemporary population-based cohort, elevated Lp(a) concentrations were associated with an increased risk of cerebrovascular events [102]. This relationship was supported not only by observational analyses, but also by genetic data, strengthening the evidence for a likely causal link. Overall, the findings suggest that high Lp(a) may represent an independent determinant of ischemic cerebrovascular risk in the general population [102]. Although important mechanistic uncertainties remain, taken together, these findings across experimental studies, population cohorts, genetic analyses, and statin-treated populations strongly support the view that Lp(a) represents a distinct and non-negligible component of coronary artery and cerebrovascular disease, thereby reinforcing its potential value as both a prognostic biomarker and a therapeutic target.

### 1.3. The Role of Lp(a) in Aortic Stenosis

Calcific aortic valve stenosis is the leading cause of valvular heart disease [103], and its prevalence is progressively increasing in Western populations, currently affecting about 2% of individuals older than 65 years [104]. Aortic valve stenosis was long considered a consequence of a passive, age-related degenerative process [105]. However, accumulating evidence indicates that Lp(a) actively contributes to disease pathogenesis by affecting aortic valve endothelial cells, promoting endothelial-to-mesenchymal transition, and ultimately driving valvular calcification [106].

Evidence from a landmark genome-wide association study (GWAS) has shown that genetic variations at the LPA locus (rs10455872), through elevated Lp(a) levels, are strongly associated with aortic valve calcification and aortic stenosis, supporting a causal role for Lp(a) in valvular disease development [107]. In line with this, elevated Lp(a) levels have been linked to both micro- and macro-calcification of the aortic valve [108,109]. Additionally, in a single genetic association study, LPA variants associated with lifelong elevation of Lp(a) were linked to calcific aortic valve stenosis independently of coronary artery disease and to early aortic valve microcalcification in first-degree relatives [110]. Of interest, the stronger effect of Lp(a) in aortic stenosis is observed in young, otherwise healthy individuals aged 45–54: in this subgroup, those with Lp(a) concentration above the 80th percentile show a threefold prevalence of valvular calcification compared to those with lower levels [10]. These data suggest that Lp(a) plays an early role in the disease continuum, before advanced calcific remodeling occurs.

### 1.4. The Role of Lp(a) in Current Cardiology Guidelines

Recent American guidelines on the management of dyslipidemia have formally established Lp(a) as a clinically relevant risk-enhancement factor within cardiovascular risk assessment [111]. In this framework, Lp(a) measurement is recommended at least once in adulthood. In individuals with features suggestive of inherited risk, such as familial hypercholesterolemia or premature coronary artery disease, cascade screening among first-degree relatives is also advised [111].

Rather than defining Lp(a) as a direct therapeutic target, current recommendations emphasize its role in refining global risk stratification and guiding the intensity of preventive strategies, with elevated levels (≥125 nmol/L or ≥50 mg/dL) acting as a modifier that should prompt more aggressive control of modifiable risk factors [111]. In patients with established atherosclerotic cardiovascular disease and elevated Lp(a) who fail to achieve LDL-C and non–HDL-C targets despite maximally tolerated statin therapy, current guidelines recommend intensification of lipid-lowering treatment with a PCSK9 monoclonal antibody in order to further reduce cardiovascular risk [111]. This approach is supported by evidence suggesting that patients with higher baseline Lp(a) levels may derive greater clinical benefit from PCSK9 inhibition, as indicated by post hoc analyses of the FOURIER trial and prespecified analyses of ODYSSEY OUTCOMES, with a potential contribution from Lp(a) reduction itself [112,113]. European dyslipidemia guidelines from the European Society of Cardiology have progressively redefined the clinical role of Lp(a) in cardiovascular risk assessment. In the 2019 recommendations, Lp(a) measurement was mainly intended to identify individuals with very high inherited levels (>180 mg/dL, ≈430 nmol/L), associated with a lifetime risk of atherosclerotic cardiovascular disease similar to that of heterozygous familial hypercholesterolemia, and was also suggested in selected cases, such as in individuals with a family history of premature cardiovascular disease or in those at borderline risk to aid risk reclassification [114]. The 2025 ESC Dyslipidemia Guidelines update further expands this framework by lowering the threshold of clinical relevance and emphasizing that Lp(a) levels ≥ 50 mg/dL (≈105 nmol/L) should be considered a cardiovascular risk-enhancing factor across the general adult population, with a continuous relationship between Lp(a) concentration and risk, thereby reinforcing its role as a modifier of cardiovascular risk beyond the identification of extreme phenotypes [115]. Moreover, the 2024 Chronic Coronary Syndromes guidelines also suggest that a single measurement of circulating Lp(a) is sufficient in individuals with suspected chronic coronary syndromes, given the predominantly genetic determination and relative lifelong stability of circulating Lp(a) levels [116].

## 2. The Impact of Lp(a) in PCI

In the era of residual cardiovascular risk reduction [117], Lp(a) has emerged as a key residual-risk factor: its plasma concentration is largely genetically determined and only marginally affected by lifestyle or standard lipid-lowering therapy [52], and the pathophysiological mechanisms already detailed make it a meaningful contributor to atherothrombotic risk [118]. As a result, elevated Lp(a) may help explain part of the persistent risk observed after technically successful percutaneous coronary intervention (PCI) [119,120]. Recent evidence indicates that its prognostic value extends beyond its established association with incident atherosclerotic cardiovascular disease and may be particularly relevant in patients undergoing coronary intervention [120].

### 2.1. Prognostic Value of Lp(a) After PCI (Figure 3)

Elevated serum Lp(a) concentrations have been linked to an increased risk of major adverse cardiac events in patients with CAD after PCI [119]. The overall body of evidence supports an association between elevated Lp(a) and worse long-term outcomes after PCI [119,120,121,122,123,124,125,126,127]. In a 2024 systematic review and meta-analysis including 15 studies and 45,059 patients, elevated Lp(a) was associated with a significantly higher risk of major adverse cardiovascular events (MACE) after PCI (RR 1.38, 95% CI 1.23–1.56) [120]. Higher Lp(a) levels were also linked to greater risks of all-cause death (RR 1.26), cardiovascular death (RR 1.58), myocardial infarction (RR 1.44), repeat revascularization (RR 1.38), and stroke (RR 1.18), although heterogeneity across studies was non-negligible and cut-off values varied. These findings suggest that Lp(a) is not merely associated with angiographic disease burden before PCI, but continues to influence prognosis during follow-up [120].

**Figure 3:**
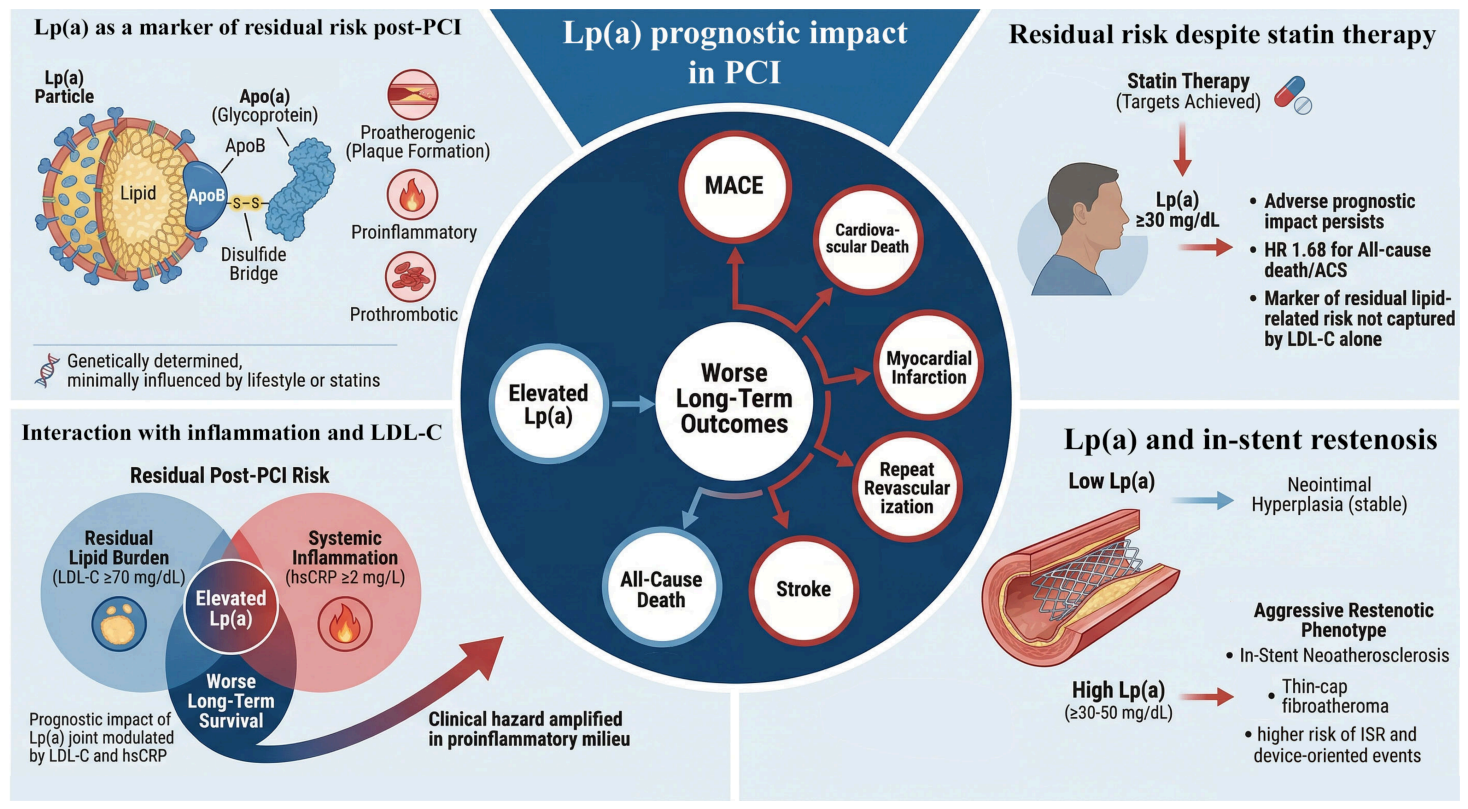
Prognostic impact of elevated Lipoprotein(a) in patients undergoing percutaneous coronary intervention (PCI). High Lp(a) serves as a marker of residual lipid-related risk despite optimal statin therapy, driving worse long-term outcomes including major adverse cardiovascular events (MACE). This risk is synergistically amplified by concurrent systemic inflammation and elevated LDL-C. Furthermore, high Lp(a) promotes an aggressive restenotic phenotype, significantly increasing the risk of in-stent neoatherosclerosis and subsequent device-oriented adverse events. Abbreviations: ACS: Acute Coronary Syndrome, Apo(a): Apolipoprotein(a), ApoB: Apolipoprotein B, HR: Hazard Ratio, hsCRP: High–sensitivity C-Reactive Protein, ISR: In–Stent Restenosis, LDL–C: Low-Density Lipoprotein Cholesterol, Lp(a): Lipoprotein(a), MACE: Major Adverse Cardiovascular Events, PCI: Percutaneous Coronary Intervention.

### 2.2. Lp(a) Prognostic Value in High Risk PCI

The prognostic signal of Lp(a) appears to be strengthened in higher-risk PCI subsets. In a large prospective cohort of 10,059 patients undergoing PCI, Cui et al. found that Lp(a) > 30 mg/dL was associated with a modest but significant increase in MACCE at 2.4 years both in the overall cohort and after propensity matching; notably, the association was more evident in older individuals and in those with left main and/or three-vessel disease [122]. Another study, by Zhang et al., analyzed a cohort of 2086 patients undergoing PCI for in-stent restenosis (ISR). The study showed that a baseline Lp(a) > 30 mg/dL was associated with a higher risk of long-term MACE compared with lower levels (26.7% vs. 21.8% at a median follow-up of 36 months; adjusted HR 1.31, 95% CI 1.08–1.58; *p* = 0.007). This excess risk was mainly driven by all-cause mortality (4.1% vs. 2.5%; adjusted HR 1.77, 95% CI 1.07–2.94; *p* = 0.03) and, to a lesser extent, by repeat revascularization. Overall, these findings support elevated Lp(a) as an independent marker of adverse long-term outcome in patients treated with PCI for ISR [123]. These observations are clinically relevant because they suggest that Lp(a) may be especially useful for refining risk stratification in patients with more diffuse, advanced, or recurrent coronary disease.

### 2.3. Lp(a) and In-Stent Restenosis

As for the correlation between Lp(a) and in-stent restenosis, in a Mayo Clinic cohort of 1209 patients with pre-procedural Lp(a) measurements, Mahmoud et al. reported that ISR occurred more often in patients with Lp(a) ≥ 50 mg/dL than in those with lower levels (17.0% vs. 11.6%), with high Lp(a) remaining an independent predictor of long-term ISR (HR 1.67, 95% CI 1.18–2.37; *p* = 0.004), particularly beyond the first year after PCI [124]. An IVUS-based study by Wu et al. involving 211 patients with stent edge restenosis (SER) undergoing PCI showed that elevated Lp(a) ≥ 50 mg/dL was independently associated with SER and with a more adverse restenotic substrate [127]. Specifically, patients with higher Lp(a) showed a greater prevalence of neoatherosclerosis (56.0% vs. 44.1%) and a lower prevalence of neointimal hyperplasia (24.0% vs. 33.8%). On multivariable analysis, elevated Lp(a) emerged as an independent predictor of SER (OR 3.39, 95% CI 2.03–5.27; *p* < 0.001). During 2-year follow-up, high Lp(a) was also associated with increased device-oriented clinical events (16.0% vs. 7.4%) and target lesion revascularization (13.3% vs. 5.1%; *p* = 0.011). Overall, these findings suggest that elevated Lp(a) is linked not only to SER occurrence, but also to a more aggressive restenotic phenotype and worse clinical outcomes after PCI [127]. Moreover, an OCT-based study of 119 patients with 125 DES-ISR lesions found that higher Lp(a) levels (≥30 mg/dL) were associated with a markedly greater prevalence of in-stent neoatherosclerosis (ISNA) compared with lower Lp(a) levels (94.0% vs. 52.0%; *p* < 0.001). In addition, lesions in the high-Lp(a) group showed a significantly higher frequency of thin-cap fibroatheroma (42.0% vs. 5.3%; *p* < 0.001), indicating a more vulnerable plaque phenotype. Overall, these findings suggest that elevated Lp(a) is strongly associated with both the occurrence and vulnerability of ISNA in DES-related ISR lesions [126].

### 2.4. Lp(a)-Derived Residual Risk and PCI Outcomes

Some cohort studies suggest that the adverse prognostic impact of Lp(a) persists even in patients receiving statin therapy or achieving apparently satisfactory conventional lipid targets after PCI. Konishi et al. showed that, among patients who had reached target lipid levels at the time of PCI, an Lp(a) concentration ≥ 30 mg/dL remained independently associated with the composite of all-cause death and acute coronary syndrome during follow-up (HR 1.68, 95% CI 1.03–2.70) [119]. In another prospective study of statin-treated CAD patients undergoing first PCI, Suwa et al. found that elevated Lp(a) levels were independently associated with a higher risk of long-term major adverse cardiovascular events (MACE), driven by cardiac death and non-fatal acute coronary syndromes (ACS) [128]. Consistent findings were later reported by Takahashi et al. in patients with acute coronary syndrome treated with statins after primary PCI: among 1131 statin-treated patients with ACS undergoing primary PCI, those with Lp(a) levels above the median value of 15.0 mg/100 ml had a significantly greater 5-year incidence of MACE (9.5% overall; log-rank *p* = 0.01). After multivariable adjustment, elevated Lp(a) remained independently associated with adverse outcomes, both as a categorical variable (HR 1.66, 95% CI 1.05–2.61; *p* = 0.03) and as a continuous logarithmically transformed variable (HR 1.35, 95% CI 1.07–1.72; *p* = 0.01). These findings support Lp(a) as a marker of residual cardiovascular risk despite statin therapy after primary PCI. [121]. Taken together, these studies support the concept that Lp(a) identifies a form of residual lipid-related risk not captured by LDL-C alone.

### 2.5. Lp(a) and Systemic Inflammation in PCI Outcomes

Another relevant aspect is the interaction between Lp(a) and inflammatory risk in patients undergoing PCI. Yuan et al. demonstrated in more than 10,000 patients undergoing PCI that both elevated Lp(a) and high-sensitivity C-reactive protein (hsCRP) were associated with a higher 5-year risk of MACCE (20.5% overall). Importantly, a significant interaction was observed between the two biomarkers (*p* for interaction = 0.019), suggesting that the prognostic impact of Lp(a) was stronger in the presence of inflammation. Specifically, when hsCRP was ≥2 mg/L, Lp(a) levels of 15–29.9 mg/dL and ≥30 mg/dL were associated with increased risk (HR 1.18 and 1.20, respectively), whereas this association was attenuated when hsCRP was <2 mg/L. Patients with simultaneous elevation of Lp(a) and hsCRP had the highest event rates, supporting the notion that the clinical hazard associated with Lp(a) may be amplified in a pro-inflammatory milieu and reinforcing the usefulness of the combined use of these biomarkers to improve risk stratification after PCI [125]. More recent data further suggest that the effect of Lp(a) may be jointly modulated by LDL-C and hsCRP [129]: Li et al. found that, after PCI, elevated Lp(a) (≥30 mg/dL) was associated with worse long-term survival over a median follow-up of 5.1 years. Of note, this effect was strongly influenced by both LDL-C and hsCRP levels. When LDL-C was <70 mg/dL, high Lp(a) was no longer an independent predictor of all-cause mortality, regardless of inflammatory status. In contrast, among patients with LDL-C ≥ 70 mg/dL, elevated Lp(a) was associated with increased mortality only in the presence of hsCRP ≥ 2 mg/L (HR 1.49, 95% CI 1.06–2.09), whereas no significant association was observed when hsCRP was <2 mg/L (HR 1.30, 95% CI 0.91–1.86). Overall, these findings suggest that the adverse prognostic impact of Lp(a) after PCI is amplified by the coexistence of residual lipid burden and systemic inflammation [129].

## 3. The Impact of Lp(a) in TAVI (Figure 4)

### 3.1. Lp(a) in the Early Development of Aortic Stenosis

Transcatheter aortic valve implantation (TAVI) has become a widely adopted procedure for the treatment of severe aortic stenosis across the entire surgical risk spectrum, with expanding indications even in asymptomatic and younger patients [130]. As its use continues to grow, the need for long-term risk stratification becomes increasingly important. The expansion of TAVI to low-risk patients, following the Evolut Low-Risk and PARTNER 3 trials, has shifted the focus from short-term procedural outcomes to long-term prosthetic valve durability and risk stratification [131]. In this evolving scenario, Lp(a) may provide incremental information for long-term risk assessment and prosthetic valve performance. As detailed in the Lp(a) pathophysiology section, the impact of Lp(a) on the aortic valve appears to be stage dependent [107]. Lp(a) has now emerged as a potential determinant of accelerated disease progression [132]. A meta-analysis of five prospective longitudinal cohorts including patients with mild-to-moderate aortic stenosis (ASTRONOMER, PROGRESSA, Ring of Fire, SALTIRE, and SALTIRE-2) evaluated individuals in whom both plasma Lp(a) concentrations and annualized echocardiographic measures of disease progression were available. The study population was relatively young (mean age 65.2 ± 13.1 years) and predominantly male (70%), reflecting an earlier stage of aortic stenosis compared with the typical elderly population undergoing valve replacement. Elevated Lp(a) levels—particularly within the highest tertiles—were associated with accelerated hemodynamic progression, as reflected by greater increases in peak transvalvular velocity and mean pressure gradient, with no significant heterogeneity observed across the included cohorts [133].

**Figure 4:**
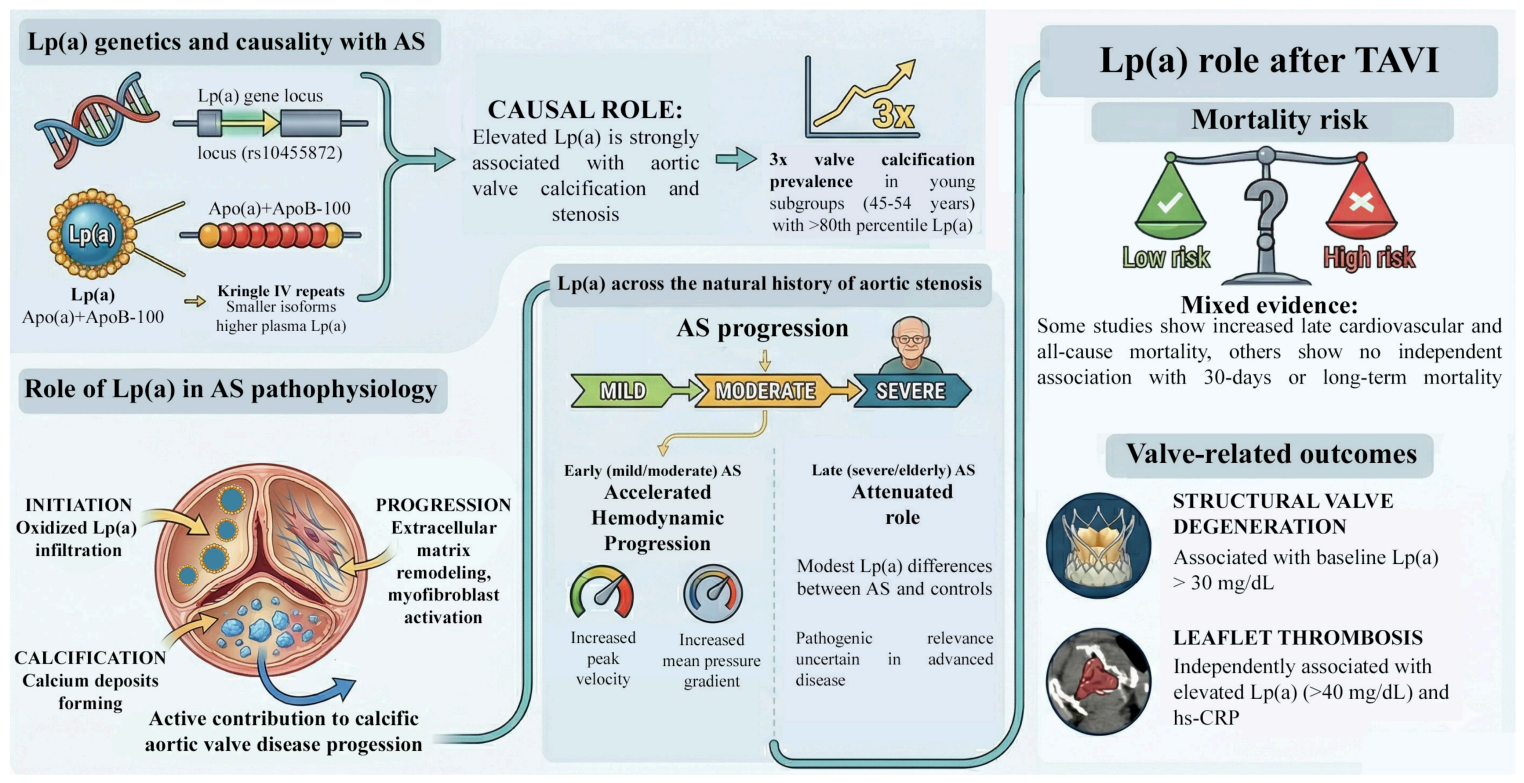
The role of Lipoprotein(a) in the pathogenesis of aortic stenosis (AS) and clinical implications following transcatheter aortic valve implantation (TAVI). Genetically determined elevated Lp(a) exerts a causal role in initiating calcific aortic valve disease through oxidized lipid infiltration and extracellular matrix remodeling. While its detrimental impact on hemodynamic progression is prominent in early-to-moderate AS stages, its relevance attenuates in severe disease. Post-TAVI, elevated Lp(a) is implicated in leaflet thrombosis and bioprosthetic structural valve degeneration, though its independent effect on overall mortality remains debated. Abbreviations: Apo(a): Apolipoprotein(a), ApoB–100: Apolipoprotein B–100, AS: Aortic Stenosis, hs–CRP: High–sensitivity C–Reactive Protein, KIV: Kringle IV, Lp(a): Lipoprotein(a), TAVI: Transcatheter Aortic Valve Implantation.

Even in the setting of lifelong elevated LDL-C, data from the SAFEHEART cohort showed that patients with familial hypercholesterolemia had a significantly higher incidence of severe aortic valve stenosis compared with non-affected relatives. In this population, elevated Lp(a) independently predicted aortic valve disease progression and the need for aortic valve surgery [134].

### 3.2. Lp(a) in Severe Aortic Stenosis

While Lp(a) has been consistently associated with accelerated progression in mild and moderate aortic stenosis, its role is attenuated in advanced disease. In a retrospective observational study comparing 503 patients with severe aortic stenosis referred for TAVI with 25,343 population-based controls, median Lp(a) levels were only modestly higher in the aortic stenosis group (20.5 vs. 18.7 mg/dL; *p* = 0.04). Patients with aortic stenosis were very elderly (mean age 80 ± 6.2 years), whereas controls were younger. The observed difference in Lp(a) levels was primarily driven by male patients (19.9 vs. 16.6 mg/dL; *p* = 0.04), while no significant difference was observed among females (22.1 vs. 21.3 mg/dL; *p* = 0.87) [135]. In line with this, a retrospective observational study included 454 patients with severe aortic stenosis referred for TAVI, stratified according to Lp(a) levels using a cut-off of ≥60 mg/dL. All patients underwent pre-procedural echocardiography, blood tests including Lp(a), and computed tomography with quantification of aortic valve calcification (AVC). Baseline characteristics were comparable between groups, although patients with elevated Lp(a) exhibited higher total, LDL, and HDL cholesterol levels. No association was observed between Lp(a) and AVC burden, nor with short- or long-term clinical outcomes. Lp(a) was not independently associated with AVC extent or mortality, including 30-day and 40-month all-cause mortality, even after sex stratification or application of alternative Lp(a) thresholds [136]. These findings raise uncertainty regarding the pathogenic relevance of Lp(a) in elderly patients and/or in patients with a confirmed diagnosis of severe aortic stenosis, suggesting that its contribution may be age-dependent and more pronounced in earlier disease stages or in younger populations.

### 3.3. Lp(a) Prognostic Value in TAVI Outcomes

Whether baseline Lp(a) levels independently predict long-term survival following TAVI remains uncertain. While some studies have reported an association between elevated Lp(a) and increased post-procedural mortality [137], others have failed to demonstrate a significant prognostic effect, highlighting the need for further clarification [136]. As for Lp(a) prognostic impact after TAVI, increasing attention has been directed toward valve-related outcomes, particularly in the context of the expanding use of transcatheter aortic valve implantation in younger and lower-risk patients [131]. In a prospective observational cohort study of 601 patients undergoing TAVI for symptomatic severe aortic stenosis (median follow-up: 3.9 years), elevated baseline Lp(a) levels (≥30 mg/dL and ≥50 mg/dL) were independently associated with increased long-term all-cause and cardiovascular mortality, as well as a higher risk of structural valve degeneration (SVD), defined as mean trans-prosthetic gradient > 20 mmHg plus an increase > 10 mmHg over time, and/or new-onset moderate or severe transprosthetic regurgitation. The association was particularly evident for late mortality, with no impact on early procedural outcomes [137]. Hypoattenuating leaflet thickening (HALT) is commonly recognized as a hallmark of subclinical leaflet thrombosis [138]. An observational study reported that elevated Lp(a) and high-sensitivity C-reactive protein were independently associated with post-transcatheter aortic valve implantation leaflet thrombosis, manifested as hypoattenuating leaflet thickening [139]. The risk increased progressively with higher biomarker levels and was greatest in patients in the highest quartile, particularly when both markers were concomitantly elevated. Thresholds of Lp(a) ≥ 40 mg/dL and high-sensitivity C-reactive protein ≥ 3.5 mg/L were proposed to identify patients at higher risk, although the clinical implications for anticoagulation strategies remain hypothesis-generating [139]. A retrospective multicenter study evaluated the association between Lp(a) levels and bioprosthetic aortic valve degeneration in 210 patients who underwent surgical bioprosthetic valve replacement and had available Lp(a) measurements. During a median echocardiographic follow-up of 4.4 years, bioprosthetic valve degeneration occurred in 15.7% of patients and was associated with significantly higher Lp(a) concentrations compared with non-degenerated valves. Elevated Lp(a) levels (≥30 mg/dL) were independently associated with an increased risk of bioprosthetic valve degeneration in both univariable and multivariable analyses. These findings suggest a potential role of Lp(a) in bioprosthetic valve durability, although prospective confirmation is required [140].

## 4. Lp(a) Relevance to Surgical Revascularization and Valve Replacement

Although this review focuses on percutaneous and transcatheter interventions, the pathophysiological substrates on which Lp(a) acts—coronary atherosclerosis and calcific aortic valve disease—are identical in surgical candidates. Consequently, Lp(a)-related residual risk is not expected to spare patients undergoing coronary artery bypass grafting (CABG) or surgical aortic valve replacement (SAVR). In the largest available cohort (18,544 isolated CABG procedures), Lp(a) was elevated (≥50 mg/dL) in 22% of patients and independently predicted all-cause death (adjusted HR 1.31, 95% CI 1.09–1.59) and major adverse cardiovascular and cerebrovascular events (adjusted HR 1.18, 95% CI 1.06–1.33), with the excess risk attenuated when at least one arterial conduit was used (*p* for interaction = 0.022)—a conduit-dependent pattern consistent with the atherosclerotic vulnerability of saphenous vein grafts [141]. Smaller long-term and angiographic series are concordant [142,143], although neutral findings have also been reported [144].

Evidence on bioprostheses is discordant. Because structural valve degeneration is a property of the leaflet rather than of the implantation route, this uncertainty applies equally to SAVR and TAVR, and published cohorts have generally pooled the two: two studies found no association with degeneration or with time to reintervention [145,146], whereas a recent echocardiographic cohort reported an independent association between Lp(a) > 125 nmol/L and stenotic/mixed degeneration (adjusted subdistribution HR 3.00, 95% CI 1.48–6.07) [147]. Lp(a) is therefore best regarded as a modality-independent risk enhancer, warranting measurement irrespective of the treatment strategy selected. To date, no randomized trial has directly compared Lp(a)-related risk between surgical and transcatheter treatment of the same underlying disease.

## 5. Emerging Therapies to Lower Lp(a) Levels

Although no therapies specifically approved to lower Lp(a) are currently available, several existing lipid-lowering agents have demonstrated secondary Lp(a)-lowering effects [148]. However, it remains uncertain whether these reductions translate into a decrease in MACE. In the specific setting of PCI and TAVI, dedicated randomized trials and well-designed outcome studies will be required to determine whether Lp(a)-lowering therapies can directly improve procedure-related and long-term cardiovascular outcomes.

### 5.1. Non-Targeted Lp(a) Therapies (Table 1)

(A) Lipoprotein apheresis

Lipoprotein apheresis is currently the most effective non-pharmacological method for lowering Lp(a). In the United States, it is FDA-approved for patients with coronary artery disease or peripheral artery disease (PAD) where diet or maximally tolerated lipid-lowering pharmacotherapy are ineffective or not tolerated, provided at least one of the following criteria is met: homozygous familial hypercholesterolemia (HoFH) with LDL-C ≥ 500 mg/dL; heterozygous familial hypercholesterolemia (HeFH) with LDL-C ≥ 300 mg/dL; or heterozygous familial hypercholesterolemia (HeFH) with LDL-C ≥ 100 mg/dL and Lp(a) ≥ 60 mg/dL [149]. Conversely, European guidelines (e.g., German guidelines) adopt a broader approach, authorizing apheresis for patients with isolated elevated Lp(a) (>60 mg/dL) and progressive cardiovascular disease, even if LDL-C levels remain within target ranges [150]. Lipoprotein apheresis can acutely reduce Lp(a) levels by more than 60%, with a mean interval concentration reduction of approximately 25–40% in patients undergoing weekly or biweekly treatment [151]. A German prospective multicenter observational study evaluated 170 patients with elevated Lp(a) levels and progressive cardiovascular disease undergoing regular lipoprotein apheresis. The patients were prospectively followed for a period of 5 years: the study demonstrated a reduction in mean Lp(a) levels (−68.1%) and a decrease in the annual incidence of cardiovascular events (from 0.58 ± 0.53 2 years before regular lipoprotein apheresis to 0.11 ± 0.15 thereafter; *p*-value < 0.0001) [152]. Despite these findings, data on apheresis in populations with elevated Lp(a)—independent of LDL-C levels—remain limited [149].

**Table 1:** Non-targeted Lp(a) therapies.

Drug	Effect	Target	Recommendation
Lipoprotein apheresis	Most effective currently available non-targeted strategy; acutely lowers Lp(a) by >60%, with mean interval reduction of roughly 25–40% during chronic weekly/biweekly treatment.	No single molecular target; extracorporeal removal of apoB-containing lipoproteins including Lp(a).	Consider only in highly selected very-high-risk patients with progressive ASCVD and markedly elevated Lp(a), especially when other options are inadequate. Not a pharmacological Lp(a)-specific therapy.
PCSK9 monoclonal antibodies (e.g., alirocumab, evolocumab)	Moderate reduction in Lp(a), generally greater in patients with higher baseline Lp(a); cardiovascular benefit is mainly driven by LDL-C lowering rather than selective Lp(a) reduction.	PCSK9.	Recommended according to standard LDL-C/ASCVD indications, not specifically for isolated Lp(a) lowering.
Inclisiran	Moderate reduction in Lp(a), broadly comparable to the class effect seen with PCSK9 inhibition.	PCSK9 mRNA.	May be used for LDL-C lowering when otherwise indicated, but not as an Lp(a)-targeted treatment.
CETP inhibitors	Significant reduction in Lp(a) in trials, likely via reduced apo(a) synthesis.	CETP.	Not recommended in routine practice for Lp(a) because this class is not commercially established and has shown limited clinical benefit overall.
Niacin	Modest-to-moderate reduction in Lp(a); less potent than newer approaches.	Hepatic apo(a) production and triglyceride synthesis pathways.	Not recommended specifically for Lp(a) lowering, as major trials did not show clear cardiovascular event reduction despite biochemical improvement.

(B) PCSK9 inhibitors

Monoclonal antibodies targeting proprotein convertase subtilisin/kexin type 9 (PCSK9 mAbs) significantly lower Lp(a) levels, with greater efficacy observed in patients with higher baseline values [148,149,150,151,152,153]. The pharmacodynamic mechanism driving this reduction is complex and multifactorial. Kinetic studies suggest that PCSK9 mAb monotherapy reduces Lp(a) production, likely by limiting intracellular apo(a) availability [154], whereas combination therapy with statins enhances the catabolism of Lp(a) particles without affecting synthesis [155]. Similarly, small interfering RNAs (siRNAs) targeting PCSK9, such as Inclisiran, have demonstrated comparable Lp(a)-lowering capabilities [156]. Although the role of PCSK9i in reducing cardiovascular risk is well documented, it must be emphasized that the primary driver is the reduction of LDL-C rather than other pleiotropic mechanisms [157].

(C) CETP Inhibitors

Cholesteryl ester transfer protein (CETP) inhibitors have demonstrated statistically significant reductions in serum Lp(a) [148], likely mediated by decreased apo(a) synthesis [158]. However, this class is not currently commercially available due to minimal clinical benefits in preventing cardiovascular events [159].

(D) Niacin

Niacin significantly reduces serum Lp(a) [160], although to a lesser extent compared to the other drug classes mentioned [148]. Its mechanism involves the suppression of hepatic apo(a) production and decreased apoB secretion via the inhibition of triglyceride synthesis [161,162]. Yet, large trials have failed to demonstrate a significant reduction in cardiovascular events in patients treated with niacin, despite observed reductions in Lp(a) levels [163,164].

### 5.2. Targeted Lp(a) Therapies (Table 2)

Given the limited ability of currently available lipid-lowering therapies to achieve substantial and selective reductions in Lp(a), the therapeutic landscape is increasingly shifting toward agents that directly suppress hepatic apolipoprotein(a) synthesis. This new class of compounds—which includes antisense oligonucleotides (ASOs), small interfering RNAs (siRNAs), and small-molecule inhibitors—has demonstrated an unprecedented capacity to reduce circulating Lp(a) concentrations by more than 90% [165,166,167,168,169].

**Table 2:** Targeted Lp(a) therapies.

Drug	Effect	Target	Recommendation
Olpasiran (siRNA)	Very potent, dose-dependent Lp(a) lowering; in phase 2, placebo-adjusted reduction reached roughly 70% to 100% depending on dose.	LPA mRNA in hepatocytes.	OCEAN(a)-Outcomes, ongoing phase 3 trial. NCT05581303.
Lepodisiran (siRNA)	Very potent, dose-dependent lowering; phase 2 showed placebo-adjusted time-averaged reduction up to about 94%.	LPA mRNA in hepatocytes.	ACCLAIM-Lp(a), ongoing phase 3 trial. NCT06292013.
Zerlasiran (siRNA)	Sustained and marked lowering; phase 2 showed approximately 81% to 86% time-averaged reduction.	LPA mRNA in hepatocytes.	ALPACAR-360, phase 2 randomized trial.
Pelacarsen (ASO)	Potent, dose-dependent lowering; phase 2 showed maximal reduction of about 80%.	LPA mRNA via RNase H-mediated degradation.	Lp(a)HORIZON, ongoing phase 3 trial. NCT04023552.
Muvalaplin (small molecule)	Oral therapy that disrupts Lp(a) particle assembly; phase 2 showed reductions up to about 82% with intact Lp(a) assay.	Apo(a) Kringle IV types 7–8, preventing apo(a)–apoB-100 assembly.	MOVE-Lp(a), ongoing phase 3 trial. NCT07157774.

The following section provides an overview of these emerging targeted therapies:

(A) Small Interfering RNA (siRNA)

Small interfering RNA (siRNA) drugs utilize short, double-stranded exogenous nucleotide sequences to silence specific genes via the RNA interference (RNAi) pathway [170]. Olpasiran, lepodisiran, and zerlasiran are siRNAs specifically designed to inhibit the expression of the LPA gene in hepatocytes, thereby preventing the production of apo(a), the key structural component of Lp(a) [171]. As for olpasiran, its role in Lp(a) level reduction was investigated in the OCEAN(a)-DOSE (Olpasiran Trials of Cardiovascular Events and Lipoprotein(a) Reduction–Dose Finding Study), a phase 2, multicenter, randomized, double-blind trial involving 281 patients with documented ASCVD and Lp(a) levels > 150 nmol/L [165]. Patients were randomized to receive olpasiran (10 mg every 12 weeks, 75 mg every 12 weeks, 225 mg every 12 weeks, or 225 mg every 24 weeks) or placebo for a treatment duration of 48 weeks. The primary endpoint assessed the change in serum Lp(a) levels at week 36 relative to baseline. Olpasiran demonstrated a dose-dependent reduction in serum Lp(a) levels compared to the control group at week 36 (placebo-adjusted mean percent changes of −70.5% with the 10-mg dose, −97.4% with the 75-mg dose, −101.1% with the 225-mg dose administered every 12 weeks, and −100.5% with the 225-mg dose administered every 24 weeks), without a significant increase in the incidence of adverse events. Building on these results, the ongoing phase 3 OCEAN(a)-Outcomes trial (NCT05581303) aims to evaluate olpasiran’s efficacy in reducing the time to first MACE in patients with ASCVD and elevated Lp(a) [172]. As for lepodisiran, a recent phase 2, randomized, double-blind, placebo-controlled trial evaluated the safety and efficacy of lepodisiran in a cohort of 320 adults (>40 years of age) with serum lipoprotein(a) levels > 175 nmol/L on stable background lipid therapy. Patients with recent cardiovascular events or significant renal or hepatic impairment were excluded [166]. Participants were randomized to receive lepodisiran at baseline and day 180 (16 mg, 96 mg, or 400 mg), lepodisiran 400 mg at baseline followed by placebo at day 180, or placebo at both intervals. The primary endpoint was the placebo-adjusted time-averaged percentage change in serum Lp(a) levels from baseline observed between day 60 and day 180. Lepodisiran achieved a statistically significant, dose-dependent reduction (−40.8% in the 16-mg group, −75.2% in the 96-mg group, and −93.9% in the pooled 400-mg groups). The ACCLAIM-Lp(a) randomized trial (A Study to Investigate the Effect of Lepodisiran on the Reduction of Major Adverse Cardiovascular Events in Adults With Elevated Lipoprotein(a); NCT06292013) is currently ongoing. This phase 3 trial is expected to enroll approximately 16,700 patients with elevated serum Lp(a) (>175 nmol/L) and established ASCVD, or those at risk for major cardiovascular events, randomized to receive lepodisiran or placebo [173]. The primary outcome is the time to first MACE. Results are anticipated in the coming years. As for zerlasiran, its efficacy was investigated in the ALPACAR-360 study, a multicenter, randomized, double-blind, placebo-controlled phase 2 study. A total of 178 patients with ASCVD and elevated serum Lp(a) concentrations (>125 nmol/L) were randomized to receive a subcutaneous dose of zerlasiran (450 mg every 24 weeks for 2 doses, 300 mg every 16 weeks for 3 doses, or 300 mg every 24 weeks for 2 doses) or placebo [167]. The primary outcome was defined as the time-averaged percent change in lipoprotein(a) concentration from baseline to 36 weeks. Zerlasiran administration was associated with a statistically significant reduction in Lp(a) values over the study period (−85.6%, −82.8%, and −81.3% for the 450 mg every 24 weeks, 300 mg every 16 weeks, and 300 mg every 24 weeks groups, respectively).

(B) Antisense oligonucleotide agents (ASOs)

Antisense oligonucleotides (ASOs) are short, single-stranded, exogenous nucleic acid sequences designed to bind to specific mRNA targets of clinical interest. They function by inducing mRNA degradation, inhibiting ribosomal translation, or modulating splicing [174]. Currently, pelacarsen is the only drug in this class undergoing clinical development. Pelacarsen is a second-generation ASO derived from APO(a)Rx via conjugation with N-acetylgalactosamine (GalNAc), exhibiting a potency thirty times greater than its predecessor [175]. This molecule selectively binds to the mRNA of the LPA gene, promoting its degradation through ribonuclease H-dependent mechanisms [176].

In a randomized, multicenter, double-blind phase 2 trial, 286 patients with established cardiovascular disease and elevated Lp(a) levels (>60 mg/dL) were randomized to receive either placebo or pelacarsen at varying regimens (20, 40, or 60 mg every 4 weeks; 20 mg every 2 weeks; or 20 mg weekly) for a minimum of 6 months. After six months of treatment, pelacarsen administration resulted in a dose-dependent and isoform-independent reduction in serum Lp(a) levels, achieving a maximum reduction of 80% in the cohort receiving 20 mg weekly. Furthermore, the drug demonstrated a favorable safety and tolerability profile [168]. The Lp(a) HORIZON trial is a multicenter, randomized, double-blind phase 3 study designed to assess the impact of pelacarsen on cardiovascular morbidity and mortality in patients with established cardiovascular disease and elevated Lp(a) [177]. A total of 8323 patients with elevated serum Lp(a) (>70 mg/dL) and a history of myocardial infarction (MI), ischemic stroke, or PAD were randomized 1:1 to receive pelacarsen (80 mg monthly) or placebo, with a minimum follow-up duration of 2.5 years. The primary endpoint—evaluated in both the overall population and a subpopulation with Lp(a) ≥ 90 mg/dL—is the time to first occurrence of a four-component MACE, defined as cardiovascular death, non-fatal MI, non-fatal stroke, or urgent revascularization requiring hospitalization. Results are expected in the first half of 2026.

(C) Small-molecule inhibitors

Unlike RNA-targeted therapies (ASOs and siRNAs), which inhibit the synthesis of apolipoprotein(a) by degrading LPA mRNA, small-molecule inhibitors represent a novel class of oral agents designed to disrupt the assembly of the Lp(a) particle itself. The leading compound in this category is muvalaplin. Muvalaplin is an orally administered small molecule designed to bind to the Kringle IV (KIV) type-7 and type-8 domains of apolipoprotein(a). By preventing the non-covalent interaction between apo(a) and apolipoprotein B-100, it effectively inhibits the assembly of the Lp(a) particle [178]. The KRAKEN trial is a randomized, multicenter, double-blind phase 2 study that assessed the safety and efficacy of muvalaplin [169]. The study enrolled 233 patients with serum Lp(a) levels ≥ 175 nmol/L and a high risk of cardiovascular events (defined as a history of coronary artery disease, ischemic stroke, peripheral artery disease, diabetes, or familial hypercholesterolemia). Participants were randomized to receive once-daily oral muvalaplin (at doses of 10 mg, 60 mg, or 240 mg) or placebo for 12 weeks. The primary endpoint was defined as the percentage change in Lp(a) levels from baseline to week 12, measured using both a traditional apolipoprotein(a)-based assay and a specific intact Lp(a) assay. The latter was employed to mitigate the risk of overestimating serum Lp(a) levels, which can occur with traditional assays due to the detection of drug-bound apo(a) fragments. Muvalaplin treatment resulted in a dose-dependent reduction in Lp(a), achieving maximum reductions of 81.7% using the intact lipoprotein(a) assay and 68.9% with the apolipoprotein(a)-based assay. Furthermore, muvalaplin therapy was associated with a dose-dependent decrease in oxidized phospholipids on apolipoprotein B (OxPL-apoB), a biomarker implicated in atherogenesis and calcific aortic valve stenosis [10,72]. Building on the promising findings from the KRAKEN study, the MOVE-Lp(a) trial has been initiated [179]. This randomized, double-blind, placebo-controlled phase 3 trial aims to enroll a cohort of approximately 10,450 patients with elevated serum Lp(a) and established atherosclerotic cardiovascular disease or high cardiovascular risk. The study is designed to determine whether muvalaplin can reduce the incidence of Major Adverse Cardiovascular Events (MACE) in the target population. Results are anticipated in 2031.

### 5.3. Gene Editing Therapy (Table 3)

Gene editing therapy (GET) enables the permanent in vivo modification of specific DNA sequences—through insertion, deletion, or replacement—utilizing either viral vectors (adeno-associated virus, lentivirus) or non-viral delivery platforms (lipid nanoparticles, electroporation) [180]. Initial early-phase trials have already demonstrated that GET can significantly reduce LDL-C levels by silencing the hepatic expression of the PCSK9 [181,182] and ANGPTL3 [183] genes. Spurred by these encouraging phase 1 outcomes and the established atherogenic role of Lp(a), some ongoing pilot studies are currently investigating GET-mediated inhibition of the LPA gene across various clinical settings, with data expected to emerge over the coming years [184,185,186].

**Table 3:** Gene-editing therapies for Lp(a).

Drug	Effect	Target	Recommendation
VERVE-301	Single-course in vivo gene-editing therapy intended to produce durable or potentially permanent reduction in Lp(a).	LPA gene in the liver.	Preclinical/research-stage investigational program.
CTX321	In vivo gene-editing therapy for durable suppression of Lp(a) production.	LPA gene.	Very early investigational development.
LPA-directed in vivo gene editing (class concept)	Potential for one-time, permanent reduction of Lp(a), unlike repeated dosing required with ASOs/siRNAs.	Genomic LPA locus.	Preclinical/research-stage investigational program.

## 6. Future Perspectives

The next decisive step in the Lp(a) field will be the transition from risk association to therapeutic validation. Several event-driven trials are now testing whether selective Lp(a) lowering can reduce hard clinical endpoints, including Lp(a)HORIZON with pelacarsen [177], olpasiran cardiovascular outcome programs [165,172], ACCLAIM-Lp(a) with lepodisiran [173], and MOVE-Lp(a) with muvalaplin [179]. In parallel, the Lp(a)FRONTIERS CAVS study is extending this paradigm to valvular disease by evaluating whether pelacarsen can slow the progression of calcific aortic valve stenosis rather than merely modify a circulating biomarker [187]. If these studies are positive, Lp(a) will move from being a sophisticated risk enhancer to a truly actionable therapeutic target in both coronary and valvular disease. A second priority will be to close the gap between biological knowledge and routine clinical implementation. The 2024 National Lipid Association update recommends measuring Lp(a) at least once in every adult, yet real-world adoption remains suboptimal despite a recent increase in testing [188]. At the same time, assay harmonization is still incomplete: the European Atherosclerosis Society has emphasized that mg/dL and nmol/L should not be interconverted using a fixed conversion factor, which remains a major practical limitation for trial interpretation, threshold definition, and longitudinal clinical use [10]. Over the next few years, broader testing, improved assay standardization, and clearer phenotype-specific thresholds will likely be as important as drug development itself. A third frontier is precision risk stratification, in which Lp(a) is likely to be interpreted less as an isolated biomarker and more as part of an integrated, clinically actionable risk profile. Although current recommendations support at least one lifetime measurement of Lp(a) in all adults, real-world testing rates remain exceedingly low, highlighting a major implementation gap between guideline aspirations and routine practice [10]. While universal screening remains the ideal long-term goal, its immediate large-scale implementation may be challenging, particularly as emerging Lp(a)-lowering therapies move closer to clinical use and prioritization of those most likely to benefit becomes increasingly relevant. In this context, machine-learning-guided approaches may offer a pragmatic interim solution. In a large UK biobank-based study with external validation in NHANES III, ML-guided prioritization of Lp(a) testing identified 26% to 253% more individuals above clinically relevant thresholds while requiring 21% to 72% fewer tests, supporting a scalable strategy to identify those at highest likelihood of clinically meaningful Lp(a) elevation and potential eligibility for future targeted therapies [189]. Beyond RNA-targeted therapies, genetic medicine may eventually open an additional chapter in Lp(a) management. Recent reviews of gene therapy and genome editing in lipoprotein disorders identify LPA among the relevant cardiometabolic targets, and preclinical data with gene-editing platforms such as CTX320 and CTX321 have shown durable reductions in circulating Lp(a) in non-human primates [190]. However, the promise of one-time genetic intervention must be balanced against major unresolved concerns, including off-target effects, irreversibility, delivery constraints, and cost. For this reason, gene editing currently represents a compelling but still early future direction rather than an immediately translatable strategy [190].

## 7. Conclusions

Taken together, the available literature supports Lp(a) as a biologically plausible and clinically relevant mediator of residual cardiovascular risk, rather than a passive bystander. Its role appears particularly convincing in the promotion of coronary atherosclerosis, recurrent ischemic events after PCI, in-stent neoatherosclerosis and restenosis, the initiation and progression of calcific aortic valve disease, and TAVI long-term prosthetic valve outcomes. At the same time, the strength of the evidence is not uniform across all settings and is currently based exclusively on observational data. For this reason, a clear distinction is warranted: the available data consistently support elevated Lp(a) as a genetically determined, lifelong contributor to residual cardiovascular risk; on this basis, Lp(a) can be used to identify patients who carry excess risk after coronary and, plausibly, valvular intervention. What the current evidence does not yet allow is any conclusion that measuring or lowering Lp(a) changes procedural or long-term outcomes: the peri-interventional and post-TAVI data remain largely observational and are limited by heterogeneous assays, cut-offs, and endpoints; no Lp(a)-specific threshold has been prospectively validated to guide peri-procedural decisions; and no completed randomized trial has yet demonstrated that selective Lp(a) reduction lowers cardiovascular events. Lp(a) should therefore be regarded, at present, as a tool for risk stratification and patient counselling rather than as a validated treatment target or a determinant of interventional strategy. For the practicing cardiologist and interventional cardiologist, several practical messages follow from this synthesis. First, an elevated Lp(a) in a patient undergoing PCI is best interpreted as a marker of inherited, non-modifiable residual risk that identifies a higher-risk profile after otherwise successful revascularization, and that argues for intensified control of all concomitant modifiable risk factors and closer follow-up rather than for any Lp(a)-directed treatment at this time. Second, in aortic stenosis the prognostic value of Lp(a) is greatest in earlier disease, where higher levels are associated with faster hemodynamic progression, and should be interpreted more cautiously in elderly patients with advanced stenosis or after TAVI, in whom competing risks and less consistent data limit its weight. Third, whether reducing Lp(a) improves outcomes after PCI, in calcific aortic valve stenosis or after TAVI, remains the central unresolved question, and its answer will depend on the ongoing event-driven trials of selective Lp(a) lowering—including Lp(a)HORIZON, the OCEAN(a)/olpasiran outcome program, ACCLAIM-Lp(a), and, for valvular disease, Lp(a)FRONTIERS CAVS—whose results are required before Lp(a) can be incorporated into procedure-specific management algorithms. In this light, Lp(a) should currently be viewed as both a risk marker and a candidate therapeutic target. It is already useful for refining cardiovascular risk assessment, but is still awaiting the outcome-trial evidence that would justify its full integration into phenotype-specific treatment algorithms after PCI, in calcific aortic valve stenosis, and potentially after TAVI.

## Data Availability

No new data were created or analyzed in this study. Data sharing is not applicable to this article.
